# Caries Decline in Preschool Children from Low Social Classes and with Migration Background in Hamburg, Germany: Outcome from Repeated Cross-Sectional Caries Epidemiological Studies

**DOI:** 10.3390/jcm11154251

**Published:** 2022-07-22

**Authors:** Ulrich Schiffner

**Affiliations:** Center for Dental and Oral Medicine, Department for Periodontology, Preventive and Restorative Dentistry, University Medical Center Hamburg-Eppendorf (UKE), Martinistraße 52, 20246 Hamburg, Germany; schiffner@uke.de

**Keywords:** caries decline, caries prevalence, caries experience, primary dentition, day-care children, socio economic status, migration background

## Abstract

Over several decades, the dental caries burden in the deciduous dentition has decreased in Germany. However, a dependency of the caries burden on social parameters, such as socio-economic status (SES) or migration background, is generally described. Therefore, the aim of the evaluation was to analyse to what extent children with a low social class affiliation or a migration background have participated in the caries decline. For the city of Hamburg, Germany, data from a series of five caries epidemiological surveys in day-care centres are available since 1977. Using the same methodology, the dmft values were determined, according to WHO criteria, and in addition including initial caries (IC). For the present evaluation, the data and the changes of caries load (caries prevalence and caries experience; dmft) over time were compared with reference to SES and a migration background. A decrease in the caries prevalence from 58.4% to 22.7% and in the dmft value from 2.6 to 0.8 was determined from 1977 to 2016 (WHO criteria). Including IC, the caries prevalence has decreased from 91.0% to 44.4%, and the caries experience shows a decline from 6.4 to 1.8. The caries reduction can be observed in children of all social classes and regardless of a migration background, although significant differences in caries prevalence and caries experience remain recognisable for each survey through 2016. In conclusion, preschool children from families with low SES or with a migration background have not been left behind in the improvement of dental health, but have also benefited from caries prevention measures in roughly the same order of magnitude as other children.

## 1. Introduction

Over several decades, a significant decrease in the caries burden has been documented for children and adolescents in Germany, as in other industrialised countries [1]. This reduction is very pronounced for the permanent dentition. In only two and a half decades, a caries decline (DMFT value) from 4.1 to 0.4 was achieved in 12-year-old children, between 1989 and 2014 [2]. In the deciduous dentition, a caries decline can also be observed, but its extent is significantly lower. Over a period of two decades, a decrease in caries (dmft) in 6-/7-year-old children from 2.9 to 1.7 is reported [3].

The positive changes in the caries burden, such as those mentioned here, are found internationally in many industrialised countries [4]. However, a social gradient of caries is also continuously documented in numerous studies [1,2,5]. As a rule, children from families with a lower social economic status (SES) have poorer dental health than children from families with a higher SES [2,5]. A similar unequal distribution of caries is constantly found when comparing children with and without a migration background. In industrialised countries, children with a migration background mostly have significantly more caries than children without a migration background [6,7]. These differences are registered for both the permanent and the primary dentition.

Whether, and to what extent, children with a lower SES or with a migration background participate in the caries decline is rarely investigated. Therefore, this paper will deal with the question, to what extent these children have also benefited from the caries prevention approaches in the course of the caries decline.

The analysis will be based on the datasets collected over decades by detailed caries epidemiological surveys in kindergartens and day care centres in the city of Hamburg (Germany). Since 1977, such scientific surveys were conducted several times, at irregular intervals, in 3- to 6-year-old preschool children using the same methodology.

The aim of the present evaluation is to examine the caries decline in preschool children in Hamburg with a low SES or with a migration background, and to compare it with the changes in children with a higher SES or without a migration background. In this way, the extent to which these children benefit from caries preventive measures will be answered. The null hypothesis underlying the evaluation is that the improvements in the caries burden in 3- to 6-year-old children do not differ significantly between the children from different social backgrounds or with or without a migration background.

## 2. Methods

In 1977, 1987, 1993, 2006 and 2016, caries epidemiological cross-sectional studies were conducted among 3- to 6-year-old children in Hamburg day-care centres [8,9,10,11,12]. The participating day-care centres were randomly selected in all of the districts of Hamburg for the first survey. These day-care centres were evenly distributed over the Hamburg city area. The subsequent surveys were conducted in the same facilities, as far as possible. If the previously selected day care centres no longer existed, or if consent to participate in the next study was not given, other day care centres in the same district were invited to participate in the study.

In each survey, the examinations took place in the respective day care centres after the parents had given their consent. The examiners had been trained and calibrated for the collection of the findings. A portable examination chair with an adjustable headrest and halogen examination lamp was used for the examinations. The caries was assessed visually, according to WHO criteria [13]. In addition, the initial caries (IC) was also registered [14]. The examinations were performed using mouth mirrors, dental probes and, if necessary, cotton rolls for plaque removal and drying. The initial carious lesions were defined as “white spots lesions” with apparently intact enamel surface or, at most, slight surface discontinuities [14].

In each survey, prior to the examination the parents were asked to provide information from which the social class affiliation of the family could be deduced. In the surveys from 1977 to 2006, the parents’ occupation was the basis for the SES allocation, and from 2006 onwards, their school-leaving graduation was used. The children were assigned to one of three social classes on the basis of the parental information. In addition, the parents were asked whether the children had a migration background.

All of the data were analysed using different versions of the statistic programme SPSS. The proportions of caries-free children and the dmft index were calculated with and without inclusion of the initial caries.

For the present evaluation, the caries values were calculated for each survey with reference to the social class and the migration background. The statistical evaluation was carried out with non-parametric tests (Chi^2^ test, Mann–Whitney test and Kruskal–Wallis test). To determine the significant differences, the *p*-value had to be ≤0.05. In order to answer the objective of the evaluation, the courses of the caries figures over the repeated transversal examinations were compared, in the cohorts of the different social classes and the children with or without a migration background.

## 3. Results

The evaluation covers a period of 39 years (from 1977 to 2016). With the exception of the 2016 study, the data are based on more than 1500 examined 3- to 6-year-old children in each survey. Table 1 summarises the development of caries prevalences and the mean dmft values, without and including initial lesions. Significant improvements in dental health can be shown over the long observation period. The caries prevalence, according to WHO criteria, decreased from 58.4% to 22.7%, and the caries experience decreased from 2.6 to 0.8 (dmft, WHO criteria). The values have reduced similarly significantly when the initial caries is included in the findings.

**Figure 1:**
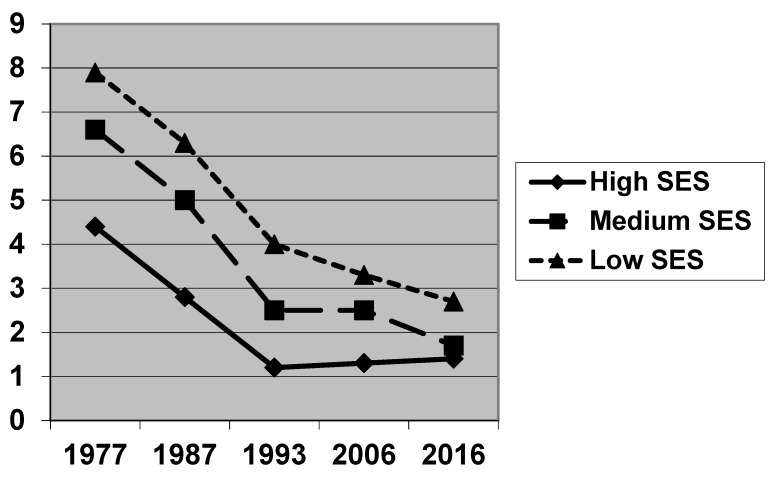
Caries experience (dmft including initial caries) in preschool children in Hamburg 1977–2016, related to the families’ socio-economic status (SES).

Table 2 shows the development of the caries burden in children from families with a different SES. For the children of all social classes, a distinct caries decline can be observed. The decline in the caries burden according to WHO criteria is particularly pronounced. Nevertheless, the significant differences in caries prevalence and caries experience between the children from the different social classes remain recognisable for each year of the study. Thus, the children from the upper social class exhibit a caries decline (dmft) from 1.6 to a remarkable value of 0.4, while among the children from the lower social class the caries reduction occurs from 3.3 to 1.9. If the initial caries is included, the caries decline is smaller, and the difference between the social classes is also smaller. Interestingly, when looking at the caries experience including initial caries, no further improvement can be shown for the children of a high SES (dmft from 4.4 to 1.4) since 1993, while the values for children of the middle and low social class have continuously improved (low social class: dmft from 7.9 to 2.9, Figure 1).

**Figure 2:**
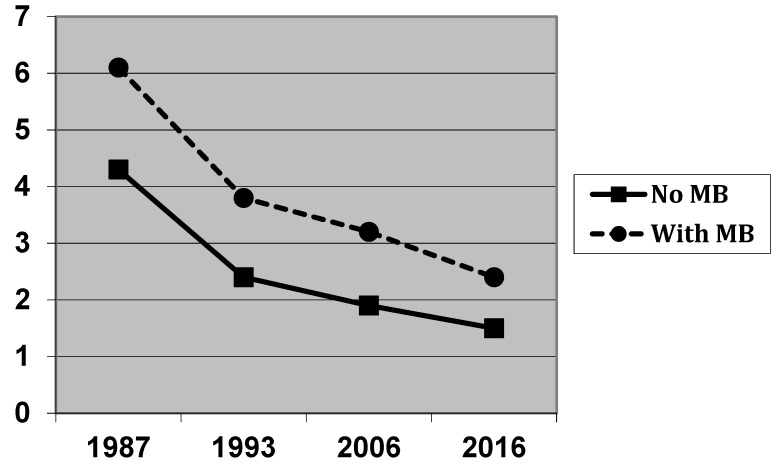
Caries experience (dmft including initial caries) in preschool children in Hamburg 1987–2016, related to the children’s migration background (MB).

Table 3 summarises the change in the caries burden from 1987 to 2016, taking into account a migration background. For all of the examinations, the children with a migration background show significantly higher prevalence rates and dmft values than the children without a migration background. However, the comparison also shows that all of the children, regardless of migration background, participate in the improvement in dental health. The extent of the improvement is roughly the same for both caries prevalence and caries experience for children with and without a migration background. When comparing the course of the dmft values over time, there even seems to be a tendency to minimize the gap between these groups (Figure 2).

## 4. Discussion

In the present evaluation, the caries epidemiological surveys conducted several times in kindergartens and day-care centres of the city of Hamburg were evaluated with a focus on the children’s social class and migration background. The evaluation documents a distinct decline in caries over almost four decades. This relates to caries prevalence and caries experience, both with and without consideration of initial caries. The caries decline can be shown for children of all social classes and independent of a migration background.

The caries registration of each survey was, starting with the first investigation in 1977, carried out according to WHO criteria, with additional registration of the initial caries. Initial caries, as defined at that time [14], corresponds to the codes one to four of the ICDAS index. The unchanged application of the same criteria over time means that the data can be validly compared over this long period of time. In addition, the fact that one investigator, in the case of the last surveys as the principal examiner, has been the same person (US) almost every year increases the validity of the results. However, changes have occurred in the course of time with regard to the basis on which an allocation to SES was made. While in the earlier surveys this was done on the basis of the parents’ profession, later their school-leaving graduation was taken as the basis. In order to be able to detect possible distortions due to this change, both of the parameters were surveyed in 2006 [11]. However, the evaluation showed that this had no effect on the study results.

The definition of migration background has also changed over the decades. Whereas this was not even surveyed in the first study in 1977, in the following studies a child was counted as a foreigner if he or she had foreign citizenship. Nowadays and concerning the last survey, in Germany a child is considered to have a migration background if he or she or at least one parent was not born with German citizenship [15]. Whether and to what extent this change of definition has consequences for the results on the caries burden in children with a migration background cannot be estimated. In all of the surveys, a highly significant statistical correlation between SES and migration background was found (*p* < 0.001), although the individuals in the respective subgroups were not completely identical.

The present evaluation proves a considerable decrease in caries in the primary dentition of preschool children over the surveys’ long period. The reduction in caries prevalence from 58.4% to 22.7% (WHO criteria) and the corresponding decrease in the caries experience, with a reduction in the dmft value from 2.6 to 0.8, document the considerable improvements in dental health. However, this statement refers to a period of almost 40 years. Within the last decade, in Germany generally, only a slight caries decline in the primary dentition is found [3]. Similarly, the figures presented here also document a strong caries decline when comparing the first cross-sectional surveys, while the caries decline since 2006 was lower, especially when initial caries is included.

The surveys were conducted in Hamburg and the results are therefore primarily valid for Hamburg. However, a national survey on oral health for three-year-old children was carried out in Germany in 2016 [3] which is the same year as the last study from Hamburg included in this evaluation [12]. The comparison of caries prevalence (Hamburg 12.0%, Germany 13.7%), as well as caries experience (dmft in Hamburg 0.4, in Germany 0.5), shows well-matched values for the three-year-olds. This can be taken as an indication that the results and developments shown here can be transferred to the national framework.

A few publications from other countries address the change in the caries burden in the primary dentition over longer periods of time. A Brazilian study, for example, documented a significant decrease in caries over 10 years in 3- to 6-year-old children. [16]. A cohort of 6- to 7-year-old children from Brussels in Belgium showed a significant decrease in caries over 15 years [17]., Observations on caries, similar to the present evaluation, in the primary dentition over 40 years are also available from Sweden [18]. Here, too, a considerable decrease in the caries prevalence and caries experience was documented in the 3- and 5-year-old children, which, however, was less pronounced compared to the development in the permanent dentition.

In many countries, a dependence of oral health on the parameters of SES and migration status have repeatedly been demonstrated [2,5,6,7,17]. This relationship is also recognisable from the present analysis. However, it is highly significant that the children from the lower social class and children with a migration background also show significantly better dental health than a few decades earlier. When comparing children of a high and low SES, the reduction of the dmft-score according to WHO criteria is, in both classes, roughly the same: in children from the upper social class 1.2 (dmft change from 1.6 to 0.4), and in children from the lower social class 1.4 (dmft from 3.3 to 1.9). The greatest improvement in the caries burden, with a dmft decline of 2.0, can be documented in children from the middle class (dmft reduction from 2.8 to 0.8).

With regard to social class, there are also indications that a certain low level of caries burden has been reached among children of the upper class, which has changed only slightly in the most recent surveys. For the middle- and lower-class children, on the other hand, further improvements in caries prevalence and caries experience are continuously discernible, up to the most recent survey. The figures presented suggest that this is narrowing slightly the gap in dental health across the social classes. Similarly, the difference in the caries scores between children with and without a migration background also gets smaller (Figure 2).

The causes of the caries decline are probably to be found on the one hand in the widespread availability of fluoride in toothpastes since the last decades of the last century [4]. On the other hand, public health measures have been taken in Germany to promote dental health at pre-school age. In 1989, the dental care programmes in day-care centres were legally established, the so-called group prophylaxis [19]. With these programmes, a public health approach was implemented, through which the children in the day-care centres are regularly informed about tooth-healthy behaviour (brushing teeth and healthy nutrition). Teeth brushing in the day care centre and dental check-ups are further components of group prophylaxis [19]. The reviews indicate an effect of the group prophylaxis on caries decline [20]. Another important step was the implementation of special dental service items (examination and fluoridation) in the catalogue of the statutory health insurance for children from the age of 30 months within the framework of individual prophylaxis, in the year 1999 [21,22].

For Hamburg, but also other federal states in Germany, the continuous increase in dental health among children from the lower social classes may also be related to the fact that the public health service visits day-care centres in socially disadvantaged neighbourhoods more often than in better-off neighbourhoods [19]. Although all of these explanations ultimately remain speculative, an influence of the aforementioned framework conditions is obvious. It is likely that all of these control measures have contributed in part to the fact that the decline in caries has also affected children from the lower social classes or with a migration background. Thus, the null hypothesis, that the improvements in dental health in 3- to 6-year-old children do not differ between the children of different social backgrounds or with or without a migration background, can be accepted.

In order to advance the existing but, compared to the permanent dentition, slow caries decline in preschool children, new dental service items were included in the catalogue of the statutory health insurance funds in 2019, which provide preventive care for children as early as 6 months of age [23]. An effect of these measures can be expected, but remains to be determined, especially since the last pandemic years have severely compromised the implementation of group prophylaxis in day-care centres.

## 5. Conclusions

In conclusion, although the caries findings of preschool children from the lower social classes or with a migration background are still less satisfactory than the findings of children from the upper social class, it can be stated that preschool children from families with a low SES or with a migration background have not been left behind in the improvement of dental health, but have also benefited from caries prevention measures.

## Figures and Tables

**Table 1:** Caries prevalence and dmft-values (both according to WHO criteria and including initial caries, IC) in preschool children in Hamburg 1977–2016.

	1977	1987	1993	2006	2016
	*n* = 1508	*n* = 1927	*n* = 1956	*n* = 1643	*n* = 933
Prevalence (%)					
WHO criteria	58.4	48.0	41.5	27.3	22.7
Including IC	91.0	74.4	52.4	50.8	44.4
dmft					
WHO criteria	2.6	2.5	2.1	1.1	0.8
Including IC	6.4	4.6	2.6	2.3	1.8

**Table 2:** Caries prevalence and dmft-values (both according to WHO criteria and including initial caries, IC) in preschool children in Hamburg 1977–2016, related to the families’ socio-economic status (SES).

	1977	1987	1993	2006	2016
Prevalence (WHO criteria)					
High SES	45.7	28.7	21.1	19.3	14.7
Middle SES	60.9	51.8	41.5	28.2	26.2
Low SES	64.9	65.8	57.9	36.9	42.6
*p* (Chi^2^-test)	<0.001	<0.001	<0.001	<0.001	<0.001
Prevalence (incl. IC)					
High SES	82.4	59.4	33.6	39.4	40.9
Middle SES	92.2	77.2	50.5	52.7	42.4
Low SES	96.4	88.2	71.3	63.6	51.9
*p* (Chi^2^-test)	<0.001	<0.001	<0.001	<0.001	0.114
dmft (WHO criteria)					
High SES	1.6	1.2	0.7	0.6	0.4
Middle SES	2.8	2.7	2.0	1.2	0.8
Low SES	3.3	3.5	3.2	1.8	1.9
*p* (KW-test)	<0.001	<0.001	<0.001	<0.001	<0.001
dmft (incl. IC)					
High SES	4.4	2.8	1.2	1.3	1.4
Middle SES	6.6	5.0	2.5	2.5	1.7
Low SES	7.9	6.3	4.0	3.3	2.7
*p* (KW-test)	<0.001	<0.001	<0.001	<0.001	<0.011

**Table 3:** Caries prevalence and dmft-values (both according to WHO criteria and including initial caries, IC) in preschool children in Hamburg 1977–2016, related to the children’s migration background (MB).

	1987	1993	2006	2016
Prevalence (WHO criteria)				
No MB	45.2	37.9	22.0	17.8
With MB	61.9	55.8	38.7	35.5
*p* (Chi^2^-test)	<0.001	<0.001	<0.001	<0.001
Prevalence (incl. IC)				
No MB	72.0	48.7	44.9	40.5
With MB	85.5	67.3	62.3	53.5
*p* (Chi^2^-test)	<0.001	<0.001	<0.001	0.003
dmft (WHO criteria)				
No MB	2.3	1.8	0.9	0.5
With MB	3.4	2.9	1.7	1.3
*p* (MW-test)	<0.001	<0.001	<0.001	<0.001
dmft (incl. IC)				
No MB	4.3	2.4	1.9	1.5
With MB	6.1	3.8	3.2	2.4
*p* (MW-test)	<0.001	<0.001	<0.001	0.002

## Data Availability

The datasets generated during and/or analysed during the current study are available from the corresponding author on reasonable request.
